# Evolution or Revolution? Recommendations to Improve the Swiss Health Data Framework

**DOI:** 10.3389/fpubh.2021.668386

**Published:** 2021-05-31

**Authors:** Andrea Martani, Lester Darryl Geneviève, Sophia Mira Egli, Frédéric Erard, Tenzin Wangmo, Bernice Simone Elger

**Affiliations:** ^1^Institute for Biomedical Ethics, University of Basel, Basel, Switzerland; ^2^Master Student, Faculty of Medicine, University of Basel, Basel, Switzerland; ^3^SIB Swiss Institute of Bioinformatics, Lausanne, Switzerland; ^4^University Center of Legal Medicine, University of Geneva, Geneva, Switzerland

**Keywords:** health data, health policy, e-health, Switzerland, biomedical research, health informatics

## Abstract

**Background:** Facilitating access to health data for public health and research purposes is an important element in the health policy agenda of many countries. Improvements in this sense can only be achieved with the development of an appropriate data infrastructure and the implementations of policies that also respect societal preferences. Switzerland is a revealing example of a country that has been struggling to achieve this aim. The objective of the study is to reflect on stakeholders' recommendations on how to improve the health data framework of this country.

**Methods:** We analysed the recommendations collected as part of a qualitative study including 48 expert stakeholders from Switzerland that have been working principally with health databases. Recommendations were divided in themes and subthemes according to applied thematic analysis.

**Results:** Stakeholders recommended several potential improvements of the health data framework in Switzerland. At the general level of mind-set and attitude, they suggested to foster the development of an explicit health data strategy, better communication and the respect of societal preferences. In terms of infrastructure, there were calls for the creation of a national data center, the improvement of IT solutions and the use of a Unique Identifier for patient data. Lastly, they recommended harmonising procedures for data access and to clarify data protection and consent rules.

**Conclusion:** Recommendations show several potential improvements of the health data framework, but they have to be reconciled with existing policies, infrastructures and ethico-legal limitations. Achieving a gradual implementation of the recommended solutions is the preferable way forward for Switzerland and a lesson for other countries that are also seeking to improve health data access for public health and research purposes.

## Introduction

Promoting the use of data and fully taking advantages of digitalisation are amongst the principal challenges that healthcare systems have been facing in recent years. With data being presented as a powerful resource [often referred to as the “new oil” (1)], there is hope that health-related information collected whenever individuals come in contact with the health system can help improve both the quality of healthcare and its cost-efficiency. For example, it has been highlighted that real-world data can be used to evaluate the cost-effectiveness of drugs after their approval or to develop more targeted therapies for cancer (2). The use of routinely collected health data can also play an important role in improving public health policies in high, middle and low income countries (3). For example it has recently been shown how health-insurance-provider data and administrative data can be used to help design the vaccination strategy against SARS-CoV-2 (4). Or else, by linking individual prescription data with data on SARS-CoV-2 infection and comparing users of Nonsteroidal anti-inflammatory drugs (NSAIDs) with non-users, Danish researchers proved that NSAIDs are not associated with increased hospitalisation, ICU admission or 30-day-mortality, thus delivering policymakers and clinician a timely answer concerning an emerging infectious disease (5). The vision of *learning healthcare* is indeed based on the idea that a beneficial circle between research and care can be achieved, if data and knowledge flow iteratively between these two integrated sectors (6). A concrete consequence of the perceived relevance of medical information to improve healthcare has been the gradual introduction of electronic health records across Europe (7, 8). Indeed, facilitating the cross-country exchange of electronic health records has also been one of the goals of recent recommendations by the European Union (9–11).

Harnessing the potential that health data offer has also been an important priority in the Swiss healthcare system. Switzerland is a confederation comprising 26 cantons (federal states) with extensive powers in the field of healthcare. This decentralisation – together with a tradition of direct democracy, the prominent role of private actors (e.g., insurance funds), and a high degree of corporatism (i.e., the involvement of interest groups in policymaking) – renders the Swiss healthcare system particularly complex (12). The complexity of the system is mirrored by the fragmentation of the health data infrastructure, which the government has been recently trying to remedy. Indeed, already in 2013 the Federal government released the “Health 2020” strategy, in which the objective of improving the health data framework of the country was transversally mentioned in the four pilasters of the strategy (13). At the same time, an ambitious effort to guarantee the creation of interoperable electronic health records (Electronic Patient Dossier – EPD) for the whole country has started, following the vision proposed in the Strategy document “eHealth Schweiz” (14, 15). The awareness that there is a need to improve the health data framework in Switzerland has been wholly present also in the research community. In 2013 the Swiss Society of Public Health published a manifesto named “Better health data for a more efficient health system” (16), which called for improving the completeness, accessibility, linkability and comparability of data concerning health. This manifesto was also endorsed by the Swiss Learning Health System, a consortium of institutions of higher education and universities aimed at strengthening the link between research and clinical practice (6). The message that the data-readiness of the country needs improvement has continued to resonate as a priority in the political sphere also more recently, as confirmed by the launch of the strategy “eHealth Schweiz 2.0” in 2018 (17) and by the new federal health policy for the period 2020–2030 (18).

Despite the constant commitment of the scientific and political fields in the last few years, the overall situation of the health data framework in Switzerland still faces several challenges. For example, data-controllers continue to express reluctance with respect to facilitating the combination of data from different sources (19). Moreover, the overall operationalisation and implementation of digital health remains in a developing phase (20). For example, the EPD was originally planned in 2007, but the adoption of the necessary legal framework was particularly troublesome (14). Even after legislation was approved, its concrete operationalisation was postponed mainly due to problems related to the certification of the institutions that manage the EPD (21). Thus, while the EPD should have been available throughout Switzerland in April 2020, as of January 2021 it is available only in one region (22). Moreover – despite the many years and the considerable funding provided – several technical questions on the EPD (such as how it will be possible for doctors to retrieve information quickly from the record) still remain open (23), thus revealing how providing an interoperable data platform in the Swiss healthcare system continues to be a challenge.

Similarly, some significant incidents during the SARS-CoV-2 outbreak revealed the need of improvement in the data-readiness of the country. At the end of July 2020, the Federal Office of Public Health (FOPH) reported how data communicated by physicians suggested that most COVID-19 infections occurred in discotheques, prior to realising that this analysis was based on a mistake with subsequent correction of the number of such infections to <2%, as compared to families accounting for almost 30% (24). Or else, in August 2020 the announcement by the FOPH of the death of a young patient due to the virus caused a sensation, but it later was revealed that the person was in fact alive and had only mild symptoms. The error resulted from the misreading of a sign in the paper-based record concerning the patient (25), which could have been prevented if data collection had been digitalised. Such examples reveal that there is still room for improvement in how health data are collected and shared between stakeholders in Switzerland, as already highlighted in a report by the OECD a few years ago (26). These persisting difficulties generate a considerable damage, in that they severely limit the health service research that could be conducted to inform policymaking in healthcare (19, 27) and they are significant obstacles to having more transparency in the health sector (12). Moreover, an under-developed and fragmented health data infrastructure represents a hindrance to the development of precision medicine within the country (28).

In this context, this manuscript presents the recommendations on how to advance the health data framework in Switzerland, which we collected in a qualitative study with Swiss stakeholders. This study is part of a broader project aimed at identifying, mapping and ordering current deficiencies of the health data infrastructure and data culture in Switzerland and the possible solutions thereto (29). The project included also a systematic review (30) and the analysis of relevant legal (31, 32) and ethical (33) issues. From the qualitative part of the project, an article on the conception of health data ownership has been written (34). In this manuscript we focus exclusively on our findings concerning the recommendations to improve the health data situation in Switzerland and we discuss their feasibility against the political and legal situation of the country.

## Materials and Methods

The overall methodology of the qualitative side of the project of which this study is part has already been previously described (34). Here we provide a quick overview and we focus on the specific methodological approach used for data analysis of this study.

### Research Team and Reflexivity

Interviews were conducted by AM and LDG, two PhD students in biomedical ethics, with previous training on qualitative research methods. The data analysis was conducted – on top of AM and LDG – by SE, a medical student, and TW and BE – both senior researchers with longstanding experience in empirical research. Given the presence of several themes in the data that concerned legal and policy aspects, FE – a lawyer specialised in data protection and experienced about the health data situation in Switzerland – was also involved in the analysis.

### Design

This study is part of a multi-stage process aimed at facilitating the harmonisation of health data in Switzerland (29). As part of this project, national experts were interviewed to identify the current barriers to health data exchange and possible solutions to address them. Since the project did not involve patients or the collection of personal health-related data, it did not need ethical approval according to Swiss regulation.^1^ Nevertheless, the local ethical committee was notified and it confirmed that ethical approval was not needed, that the project respected general ethical and scientific standards and that it could thus proceed (EKNZ req-2017-00810).

### Settings and Data Collection

Experts were selected based on purposive sampling combined with snowball sampling. Purposing sampling is an established sampling strategy in qualitative research which consists in “selecting “information rich” cases, that is individuals, groups, organizations, or behaviours that provide the greatest insight into the research question” (35). A first list of potential experts was drafted based on literature analysed for the systematic review (30) that was also conducted as part of the project. Potential experts to be interviewed were divided according to their occupation and/or expertise into three categories: (1) researchers working on projects of national importance which involved the collection and sharing of health data from different sources; (2) policymakers and public officials involved in the health data framework; and (3) directors or administrators of institutions having a health database. Experts from the initial list were contacted via email by AM and LDG, who explained the purpose of the study and asked for availability to be interviewed. Those experts who were eventually interviewed were also asked for further recommendation as to other stakeholders that they recommended to interview (snowball sampling). In total, out of the 58 experts who were contacted, 48 agreed to be interviewed, whilst the remaining either declined or did not reply. Interviewees included 28 researchers with experience in merging health data from different sources in Switzerland, 10 individuals from policy or administrative bodies involved in the steering of health data policy (e.g., from the Federal Office of Public Health or Federal Statistical Office) and 10 stakeholders of other health databases (e.g., disease-specific registries, cancer registries, hospital databases or private health databases). Often experts covered – or had previously covered in their career – more than one role. Experts were interviewed in person or via skype/phone, according to their preference. Whereas, the majority of the interviews took place in English, some experts were interviewed in Italian, German or French – official languages of the Swiss Confederation. The interviews lasted between 38 and 131 min, and the majority (39/48) were one-to-one. Interviews were conducted between May 2018 and September 2019 depending on experts' availability. Experts consented to participate in the study, for their interview to be recorded and transcribed verbatim, but eliminating reference to personal attributes that could lead to identification.

### Qualitative Analysis

For this manuscript we relied on Applied Thematic Analysis as described by Guest et al. (36) Transcribed interviews were initially analysed by AM, LDG and TW, with the objective to identify overarching topics and to divide the transcripts in segments related to those topics - a process which Guest et al. define “segmentation”. After this process, for this manuscript we considered only the segments that related to the overarching topic of “recommendations.” These were mostly related to the last section of the interview guide we used in our project (see Supplementary Material), in which we asked participants to provide recommendations to improve the Swiss health data landscape. Moreover, given the semi-structured nature of our interviews, additional segments containing recommendations were also present in other parts of the interviews (e.g., when participants were asked if they encountered legal or ethical challenges in their work and if they saw room for improvement). The segments containing recommendations were grouped in a new database and the following procedure was followed for analysis. First, half of the segments were read and manually annotated by AM and the other half by SE, with the objective of identifying thematically-related recommendations. The annotations were then discussed between AM, SME and TW, and a tentative coding tree – i.e., a list of codes encompassing the meaning of the different recommendations – was developed. Thereafter, a codebook was developed to help define the boundaries between codes, as recommended by Guest et al. (36) The codebook included for each code: (1) a brief one-line intuitive definition of the code; (2) an extended and more detailed definition; (3) some notes highlighting when to use the code and when not to use it; (4) an exemplary segment for the code in question. With the coding tree and the codebook, the segments concerning recommendations were then finally assigned to the different codes by AM and SME. Each of these two authors coded half of the segments, and then checked the other half to ensure inter-coder agreement. Segments for which coders were in disagreement were discussed collectively between AM, SME, LDG, and TW until unanimous consensus was reached on the code to which the segment should be assigned. All the authors then revised this analysis, elaborated the systematisation of the codes and their organisation into categories and discussed the implications of the recommendations made by the interviewees.

## Results

**Figure 1:**
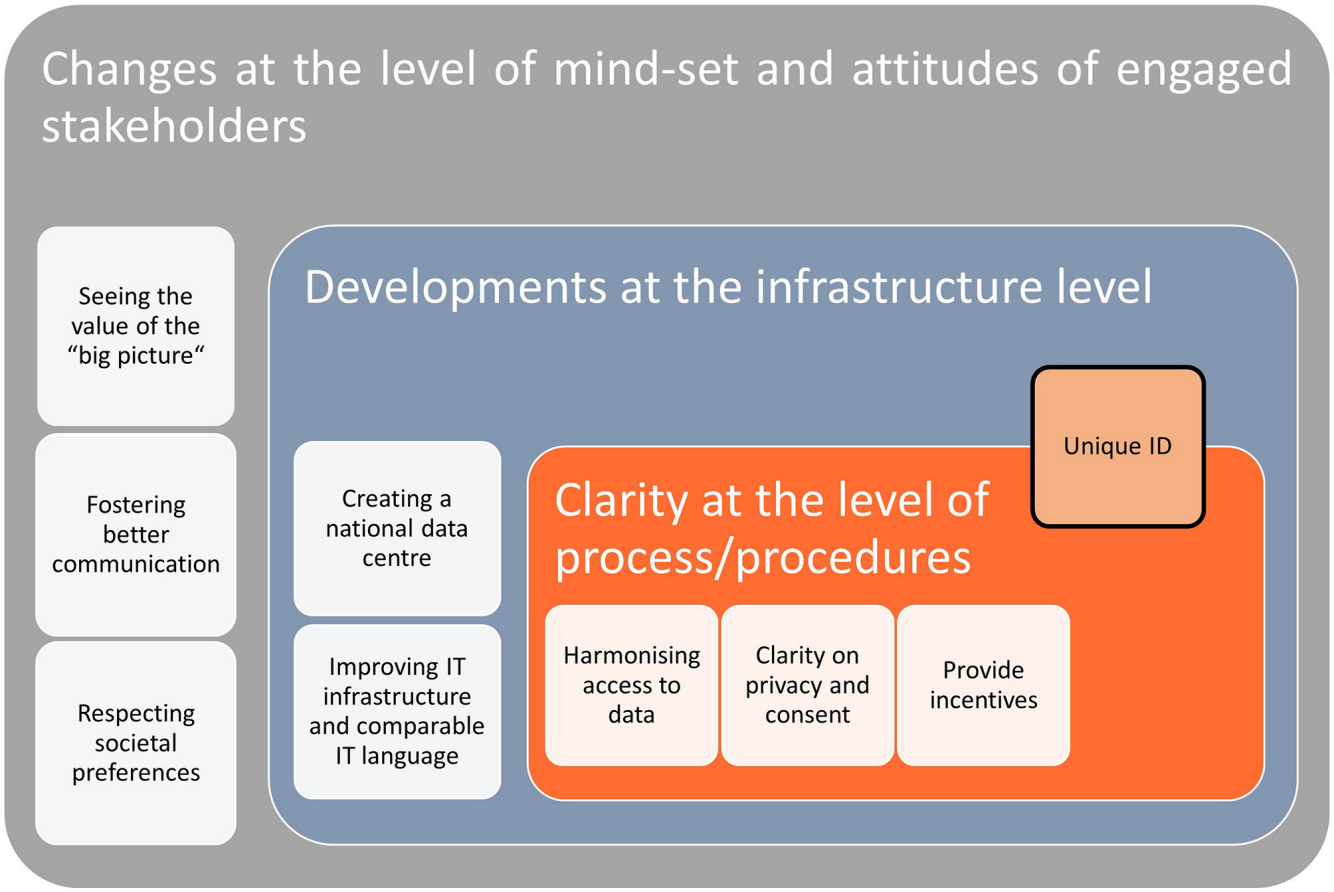
Overview of the recommendations and their different levels.

In the interviews, a series of recommendations that covered different topics and suggested different solutions were present. First, some recommendations concerned how to change the general orientation and the mind-set that lay behind the governance of health data in Switzerland. Second, other recommendations targeted more concrete developments that can be undertaken with respect to the health data infrastructure. A third set of recommendations were aimed more specifically at suggesting how to improve clarity of the processes and procedures concerning access or exchange of health data. The categories and sub-categories of recommendations are summarised in Figure 1 and then presented in more details in the sections below. All quotes presented below have been cleaned (e.g., by eliminating repetition and grammar mistakes present in the recording) and those which were originally in French, Italian or German have been translated into English.^2^

### Changing Mind-Set and Attitudes

A considerable amount of recommendations proposed by the interviewees highlight some changes that should be adopted by various stakeholders in how they approach the issue of health data processing and sharing, as summarised in Table 1.

**Table 1:** Recommendations concerning Mind-Set and attitudes.

**Recommendation**	**Concrete implications**
Seeing the value of the “big picture”	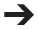 a clear health data strategy must be developed: this includes agreeing on the important objectives that need to be achieved by collecting and processing health data.
Fostering better communication	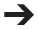 ensure that different actors are continuously engaged in a proactive exchange: institutions and initiatives in the health data framework have to be known by all actors to coordinate efforts.
Respecting societal preferences	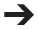 the development of the health data framework has to combine efficiency with considerations for population preferences and attitudes.

First, one urgent concern expressed by the interviewees is that progress in the health data framework requires various stakeholders involved to think long term about the purposes and the reasons for data collection and data sharing. Having long term plans on the objectives that are to be achieved by the analysis of health-related information is thus conceived as a condition to stimulate the improvement of the health data situation in a coherent way and without wasting resources.

“*The first reason is that data without a scientific question are useless. And I think that Switzerland needs to ask [.] the question of: “What will these data be used for?”. […] If there is no scientific question, there is a lot of data being collected which are useless. And a lot of data which would be useful, which are not collected”* (Res20^3^).

The importance of having a clear plan and a clear overarching idea of the final purpose as to why health data are collected is necessary for more concrete actions – such as defining standards for data collection. This element of having a “data strategy” in Switzerland was noted as such:

“*Maybe the problem is not that there is no data. The problem is that there is no consistency about the data existing. I think we could play a big role in trying to make structural data ready for use. But for that it has to be simplified somewhere else. (gathers thoughts).a data strategy, so far as I know, is not existing in Switzerland”* (Pol5).

Second, interviewees expressed that fostering communication and collaborations between the different actors involved in the health data framework is an important step to improve the situation. In the following segment, one expert mentioned the need for a much more open dialogue.

“*Participant: Those efforts (referring to initiatives working with health data) should know from each other and there should be some national exchange across those efforts (mentions the names of different initiatives)*.

*Interviewer: I see. So more collaboration between all these different actors?*

*Participant: Yes…and not necessarily collaboration. I think an exchange of information would already help. I mean just to know from each other. Maybe some informal meetings”* (Res2).

Exchange of information concerning, for example, the health databases already available, their content and their potential would avoid the creation of so-called “data cemeteries”. That is, such exchange of information can address the problem of underuse of available health data.

“*Well, for a researcher I would say: “Before starting to collect data, look around what's available.” Because, ok, there is the issue of “open data”: everybody wants “open data,” but then there is some - how to say – “schizophrenia” out there. Everybody wants open data, but nobody seems to use it. We were the first to use the data of our hospital. Nobody knew how to extract it. So we took 6 months with the informatics team to know how to extract the data. Now they have a team that only does that, but we had to start it. There might be lots of data, what we call - what I call – “data cemeteries” out there, with data that might be suitable for your research”* (Res22).

The third series of recommendations in terms of general attitudes is that stakeholders working in the field of health data should work in a way that holds in high respect the preferences of the society in which they are active, their concerns and their priorities. With respect to societal preferences, some experts hinted at the importance to respect more specifically certain features of the Swiss society when designing the development of the health data infrastructure.

“*Switzerland is Switzerland. And Switzerland is very decentralised and therefore also the databases are accordingly decentralised. Now, what we could offer is (to have a) centralised (solution), but where data is collected in a decentralised way”* (Pol4).

One expert also mentioned that these specificities of the Swiss approach could also have positive upsides

“*In one vision, you can say it (Switzerland) is a fragmented system. You have basically the three levels: the federal level, the cantonal level and the communal level, which are the three acting levels with different characteristics and competences. […] So for me, looking at the global challenges we are facing in the field, I think that it's a serious advantage to be in a decentralized system. [.] It's not a surprise that Switzerland is currently becoming a very important place for blockchain. It's because of this strong, cultural and decentralized distributed approach of the people here and the way they see the world”* (Res26).

### Developments Necessary at the Infrastructure Level

Many experts addressed in their recommendations the fact that the health data infrastructure should be improved from many points of views, as presented in Table 2.

**Table 2:** Recommendations concerning infrastructure.

**Recommendation**	**Concrete implications**
Create a national data centre	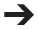 create an institution or an organisation that is capable of coordinating and combining the requests for data access and data linkage for the healthcare and research sector.
Improve IT infrastructure and promote comparable IT language	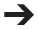 invest on a IT infrastructure that allows an effective reuse of health data. Also, ensure that data from different datasets are compatible by promoting the use of standard nomenclatures and formats
Unique Patient Identifier	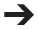 in a decentralised system like Switzerland, a unique identifier to link data concerning the same person from different sources should be enabled.

In this respect, a series of suggestions proposed the creation of an institution resembling a “national data center” in charge of managing and coordinating the different data sources available in Switzerland. Although all the features that such an institution could have and its exact architecture were not described in details, it was mentioned that one key characteristic it could have is to allow linkage of data from the different sources.

“*That's why I would actually say we need a center which is allowed to link data and for the linkage you need everything which is identifying. And once the link exists, we can attach the research data and the researcher or whoever who wants to do the analysis”* (Res14).

Not only would this data center facilitate linkage, but it would also facilitate sharing, in that it could evaluate the request of access to different types of data in a systematised fashion.

“*There needs to be some umbrella (organisation) where you can put the data and share it just to people that have a really good research question and you have maybe some sort of a process in place how you approve those data sharing processes”* (Res7).

Other recommendations suggested that the health data infrastructure should be advanced. This would entail, for example, aligning the different clinical information systems that hospitals use. According to one expert, improving the IT systems of the country would be feasible since the technical expertise in Switzerland is present.

“*I also would think that actually it would be smart if all the hospitals have the same clinical information systems, the same place where they collect [their] dataset”* (Stak6).

“*And then of course, we have to resolve a lot of logistical problems you know…the IT systems…but I think they are all solvable honestly. […] We have a lot of good IT guys in Switzerland. They know what computers are, they can do that.and I think their ideas will somehow work”* (Res23).

Expanding the IT Infrastructure in itself should however not be the only concern. One expert specifically addressed the need to align standards as well, when reflecting on the idea of creating new health registries. Similarly, other experts also mentioned the need to work on promoting comparable IT languages in the infrastructure which are already present and those which will be built.

“*At the same time, registers are in many cases the only instrument to obtain quasi real-life evidence, aren't they? […] And the question will be then: “Must you really for every question, for every sector, for every (medical) intervention – now I am thinking really about the future – must you really then for every single thing in the future create a register? “ And then also maintain it and carry it forward. Or would it not be (possible) also with a strong standardisation of health data at the source?”* (Pol6).

“*So you have to unify, to finally unify the semantics. And then you have of course to to find a way to code data that they are shareable. They are just not shareable right now”* (Res13).

Improving and harmonising the semantics of how data are collected would allow, to some extent, to combine existing IT infrastructure without the necessity to “revolutionise” the system. One expert made this point referring to the specific example of the electronic health record.

“*Yeah, I think realistically right now we can't ask every major Swiss hospital to use the same electronic health record. We can't. […]. But what we could do is build a sort of under-scaffolding. So you have all the Swiss Hospitals with their different electronic health records but like we are piloting here in (Swiss city) with this, kind of behind-the-scenes, behind the façade, you could build these common language, common electronic language […].you could create a common language …a common - you know - electronic processing language where you harmonize all these data, all these clinical data”* (Res1).

Many interviewees also recommended that a system to efficiently link health data from different sources should be implemented. This type of recommendation concerns both the infrastructure level (since linking requires the appropriate technical support) and processes/procedures (since linking operations also need to follow appropriately regulated protocols). In this respect, it was highlighted that the ideal situation would be that of having a Unique Patient Identifier (UPI) for patients, so that every time data are recorded about the same person, they can be combined with data of that very patient from other databases. The use of such number would naturally have to be properly regulated.

”*Yes, just each person has this number and every time you go to the doctor, you go to Spitex (a form of intermediate care offered in Switzerland), you go anywhere this is registered and you can link it. But I think it needs restrictions on who has access to this, because it's very sensitive data. This has to be dealt with. And it needs some centralised place where this linkage is done.”* (Res9).

“*So I think we spend too much money and time (laughing) with single different solutions (to do the linkage) and it would be also time in the health sector to get there a general way of using this [unique identification] number, a safe general way to have this number used”* (Pol3).

“*Well (laughs) it could be much easier as for example in the Nordic countries where you can track all the…where you have the information from the whole health system together or more or less together and identifiable”* (Res6).

### Clarity at the Level of Processes and Procedures of Data Access

Several recommendations expressed by the experts did not focus on attitudes or generally on the infrastructure level, but they addressed more specifically improvements that could be made with respect to the mechanisms necessary to access and/or share the data (see Table 3).

**Table 3:** Recommendations concerning processes and procedures.

**Recommendation**	**Concrete implications**
Harmonise access to data	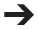 ensure that access procedures to data are less fragmented and dispersed, to facilitate the identification of data sources and the transparency of the process to obtain access to such data.
Clarity on privacy and consent	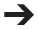 educate researchers on the data processing legal rules and implement more broadly a simplified pathway to allow the reuse of health data with more relaxed consent requirements.
Provide incentives	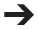 create tools to favour the cooperation between the different institutional actors that need to collaborate in the fulfilment of the procedures for data sharing and access.

In this respect, an important recommendation referred to the need of streamlining and harmonising the concrete procedures of how data are accessed. This would entail, for example, making it clear and easy for researchers that have a project idea to know who they have to approach in an institution to inquire about the data available in such institution.

“*Harmonisation of processes would be that every hospital, for example, implements a consulting group where a researcher can go to if he has a project in mind that he would like to request data for”* (Stak3).

In some cases, experts highlighted that harmonising access to data would also require trying to implement regulations that streamline access procedures to data, possibly with the creations of step-by-step procedures on how to collaborate in the sharing of data. In this respect, there were calls for uniformity of how regulations are practically applied, rather than a call for adding or changing the law per se.

“*I would never want more regulations because that increases complexity. What I would like is more for people like me, clinical researchers, to have a very simple guide and very simplified information that we can look at when we are on the verge of doing such a collaboration. Even having a platform with very clear and simple steps and having the tool to share data. That would be very helpful”* (Res17).

“*I wouldn't call it more regulations. I want just to have that the true regulations are always applied in the same way. And then if we see that certain types of projects are impossible - truly impossible - in Switzerland, then probably you need to change regulations”* (Res10).

Accessing data requires not only compiling forms and following procedures, but also complying with data processing rules and consent requirements. The need for more clear legal provisions concerning data processing rules was highlighted, for example, with respect to healthcare service research with already existing data.

“*As I said…I think the research in Switzerland.the opportunities for research, particularly for research with existing data are very narrow already. So… yes a framework that allows more and gives clear rules for this more, it makes sense of course”* (Res3).

With respect to the role of informed consent, it was argued that the possibility of using the data without the explicit permission of the patient – maintaining however the possibility for them to opt-out from processing – should be more broadly implemented.

“*I think one is to sort out the consent process. Ideally, really ideally to cancel the “opt-in” (i.e., that explicit patient consent is necessary to process data) and to go for an “opt-out” as other European countries do* (Res13).

There was also awareness that another way to improve the clarity of the rules on data processing and its interaction with privacy is to improve the education of data processors (e.g., researchers).

“*Participant: Maybe we can improve.the curriculum of the researchers or health professionals so that they can really know about what are the data, how they can share them, what are the legal frameworks*.

*Interviewer: So more like offering some training to these researchers*.

*Participant: yes, training is very important. Because when you train people early, they don't make mistakes afterwards”* (Res18).

More in general, a sense that some of the ways how ethics has been traditionally implemented into research should be re-thought – e.g., by clarifying more specifically for which research projects involving only the use of data (e.g., retrospective registry-based studies) ethical approval is not required. This would entail – as recommended by one participant – to revise the balance between privacy for the individual and the benefits that can be produced for society if health data are more easily usable.

“*Well, I think that it would be very helpful to legally implement the value, the ethical principle that also the interest of the public can overrule the subjective rights […] under certain circumstances.[…] and I think that if we look at other countries such as Great Britain or Norway, Sweden, we could really learn a lot from them on their way to collect [health data]on a population base”* (Res4).

Lastly, a few experts mentioned also that incentives should be provided to ensure that the procedural work and the collaboration between different institutional actors in the health data framework are carried out. Such incentives could be, according to one expert, of a financial nature – e.g., to ensure that Swiss hospitals harmonise their health records.

“*If you could require Swiss hospitals to do this [harmonise their health record systems] and you have to give them money of course to do this. Because this would be a big job and then with this undue layer of harmonized data, that all have the same meaning and speak the same language.you could create a pathway to share these data”* (Res1).

The director of a health database highlighted that incentives for institutional actors to perform all the procedural chores necessary for exchanging data could also be of a political and legal nature.

“*It would be necessary - in my opinion - to have instruments that make such obligatoriness (to collect certain data) a true obligation. […] We (as a register) are not capable of going around Switzerland and say: “You have not send us your last data, you are lagging behind.” But we should have the instruments that allow to apply or that permit// some instruments that could be legal or else, that would allow these data registries to work well”* (Stak1).

## Discussion

As the debate to promote a better use of health data is still a priority in the Swiss political agenda, our study presenting recommendations on how to improve the health data framework can provide valuable insights on how to reform this sector in the future. Here we analyse experts' recommendations against the policy, societal and legal background of Switzerland to reconcile some of the swift changes proposed with the existing context of the health system in Switzerland.

A relevant finding from our interviews is the focus of many recommendations on changes that are needed in the mind-set and attitudes of the actors involved in the processing of data. Although strengthening the data infrastructure in health remains crucial (26), it is important to also secure the commitment of stakeholders, whose approach and mind-set are preconditions to develop and exploit the health data infrastructure. For example, in a study on the development of a national programme for information technology in the public health system of the United Kingdom, it was noted that “persuading” stakeholders to commit to the development of the health data framework – which was likely to produce substantial benefits only in the long run – “is at least as great a challenge as the technical one” (37). Lovis et al. made a similar point when reflecting on the Swiss situation and emphasised that “the success of eHealth projects depends on many factors besides purely technical aspects” (38). A potential solution could be that of selecting specific areas of the healthcare sector where the data infrastructure should be reinforced and where the use of data should be facilitated, and provide long-term financial incentives for such areas. For example, the Swiss Cancer Research (the most important foundation for cancer-related research in the country) has launched since 2016 specific funding for the development of projects in the field health-service research, to incentivise research relying principally on the secondary use of routinely collected data (39). Providing specific funding for projects using and/or reinforcing the health data landscape in specific areas can however also be perceived as unfairly advantaging researchers with expertise on that area and indirectly limiting the freedom to pursue research in different and uncoordinated topics.

Our interviews also highlighted that having an explicit and long-term health data strategy for Switzerland - and defining what specific achievements are to be reached with such strategy – could help in changing the mind-set of relevant stakeholders. In fact, another qualitative study evaluating Canada's e-health policy underlined the importance of having a comprehensive and well-structured national strategy to favour the development of information technology in health, underlining in particular the need “to align the investment in information technology with the priorities of the health care system and of health care providers in order to accelerate adoption and achieve early return on the investment” (40). Switzerland formally has a national e-health strategy, but this is almost exclusively focused on the introduction of nationwide interoperable EPD that needs to be offered – at least initially^4^ – only in the in-patient sector (hospitals and nursing homes) (17). Such “narrow” approach in the e-health national policy can have positive side effects, such as setting more specific objectives and parameters, rather than turning the e-health policy in a collection of general political statements (41). However, a “narrow” approach also runs the risks of not providing a clear long-term vision for the evolution of the whole health data framework: in the digitalisation of healthcare, the EPD is a good start, but cannot represent the final objective (42). Moreover, the lack of a more comprehensive health data strategy could lead to ineffective multiplication of efforts. Indeed, in Switzerland there are several national initiatives aiming at improving the health data framework: firstly, the Swiss Personalised Health Network (SPHN), a consortium supported and financed by the Swiss Federal Government and other important institutional partners with the objective of promoting personalised medicine through a better use of health-related data (43); the EPD project mentioned in the introduction; the Swiss National Cohort (44); SantéPerso^5^ a project focussed on precision medicine; the Swiss Data Science Center^6^ developed by federal universities to bridge the gap between data science, academic research and industry; or also innovative citizen-science projects like MIDATA, a platform organised as a cooperative through which individuals can make their data available for further use.^7^ However, there is little formal coordination between these initiatives (45), which could be detrimental in the long run. This limited coordination is linked to another set of recommendations expressed in our interviews, i.e., to favour communication between the different actors, who are now independently pursuing diverse efforts aimed at improving the health data situation. The SPHN recently received an additional 66.9 million CHF (equivalent to 62 million euros) of funding for the period 2021–2024 (46), and could thus take a leading role in this respect, by continuing the efforts recently initiated and by enhancing the visibility of the solutions that it is proposing (see below).

At an infrastructure level, not only a general need to improve the IT infrastructure was expressed in our interviews, but also a more specific proposition emerged. Participants insisted that a supra-institutional centre responsible for managing the procedural steps necessary for access and/or linkage should be created. Efforts in this sense are already in the pipeline: for example, the recent reform of the Cancer Registration Law has led to the creation of a national coordination center (the National Agency for Cancer Registration) operating in coordination with a national center for epidemiological research (the National Institute for Cancer Epidemiology and Registration) (47).^8^ Their competence is limited to health data concerning cancer for the moment, but Art. 24 of the same law (48) opens up the possibility to use this legal framework to also record data concerning other non-transmissible widespread or dangerous illnesses in the future. On a similar line, the Federal Statistical Office (FSO) has been recently developing an internal office to perform linkage for third-parties (e.g., researchers) between external data from different sources and/or data from the FSO itself.^9^ This represents a form of standardisation – indeed a standardised form for applying to this service was developed (49) – and centralisation, since the service represents a unique point-of-entry for the whole country. However, both the Cancer and the FSO coordination centers are too narrow in scope as compared to the idea of a national data center envisioned by our participants. Given the de-centralised nature of the Swiss healthcare system and the fragmentation of data sources, the development of a national data center should not entail the transfer of data ownership and a centralisation of data, but it should rather act as a one-stop-shop structure to access the different sources of medical information in the country. This would facilitate the possibility of combining data coming from different institutions and help reduce – together with appropriate incentives – the fragmentation of access procedures to data (see also below). An effort in this sense is underway as part of the SPHN, which led to the adoption of a semantic interoperability framework between university hospitals^10^ and is also currently creating a *Federated Query System* that would allow researchers to quickly verify what data are available according to certain search criteria within the datasets of different university hospitals (50).

Creating a national data center is connected to the further recommendation of establishing a UPI to be transversely used every time health data from the same person are collected. The presence of such an identifier would facilitate the linkage of data from different sources, which now is often done by probabilistic linkage methods – often used effectively in the Swiss context (51), but which have some inherent limitations (52). Recording data through a UPI is common in New Zealand (53) and especially in the Nordic countries, where the UPI has proven to be very useful to facilitate health services research (54, 55). For example, in Denmark every resident is assigned through the Danish Civil Registration System a UPI, which is then also used to record peoples' health data in virtually every database, thus allowing accurate linking and facilitating registry-based research (56). In Switzerland, the newly created EPD foresees the creation of an identification number assigned to the record of each patient.^11^ This number is *derived from*, but also *different to* the Social Security Number normally used by citizen (e.g., for tax purposes, or to buy health insurance): connecting the EPD directly with the Social Security Number was initially planned, but then ruled out for legal reasons and for fears of potentially compromising citizens privacy (57). As a consequence, the identification number used for the EPD represents a step toward a *universal* UPI, but presents several drawbacks. First, it is difficult to exploit the linking possibilities offered by the EPD and its identification number, since the secondary processing of EPD data for research purposes could prove very controversial as the EPD was built on the idea that it would only be used for care purposes.^12^ Second, the EPD identification number would cover only data recorded in the EPD itself. As offering the EPD is currently mandatory only for hospitals and nursing homes (the latter starting from 2022) and data are saved in a PDF format, there is a risk to miss out data from the outpatient sector and to have data structured in a way that make many analyses very difficult (58). Moreover, differently from countries like Estonia and Denmark where an electronic health record is automatically created (59), participation in the Swiss EPD requires the explicit consent of the patient, who can also freely decide to eliminate or hide any of the information therein recorded. To really favour the secondary use of data, a clear legal basis for the use of the same number to record and link data from the EPD and other data sources would be a necessary step. This would require adequate ethical and security measures that are approved by the population, as expressed by the recommendation to follow societal preferences. Indeed, improving health data access necessarily requires to “engage with [its] underlying political, human, and legal challenges” (60), since neglecting societal preferences, fears and hopes might backfire. This happened, for example, in Iceland when the government tried to introduce a new system of health data registration/linking (61). In this regard, it is important to note that – in the last few years – the willingness to have health data saved electronically and the trust that the institutions collecting health data respect privacy have both diminished in Switzerland, despite remaining generally high (62). The origin of such decreased trust should be investigated.

At the ethical and regulatory level, another important set of recommendations concerned the clarification of the role of consent and of data protection legislation for the processing of health data. Swiss legal and ethical standards already provide for a research exemption (i.e., special rules for data processing for research or statistical purposes) and for exceptions concerning consent, which allow the secondary use of data through general consent (e.g., a consent covering broad areas of research, rather than simply one specific project) or even without consent in some cases (31). However, there is disagreement in the Swiss legal field to what extent these exemptions can be implemented in practice without compromising individual rights (63, 64). Moreover, the fact that experts recommended clarifications on such topics shows that – although these exceptions exist in the letter of the law – their concrete implementation still necessitates improvements. In particular when data are combined from (or linked between) different sources, many questions about the actual operational functioning of the law remain open (65). For example, the legal conditions for using medical data routinely collected by health insurances to conduct health services research theoretically exist (66), but it is unclear how to design such research projects in a legally compliant fashion. Uncertainty on the concrete operationalisation of legal rules concerning the reuse of data can create a catch-22 situation: when researchers plan studies involving the secondary use of data from other institutions (e.g., health insurances), “funding agencies routinely request a guarantee that data access should be possible, while data owners [from those institutions] often may not be able or willing to give such a guarantee [until] funding is available” (45). To help solve these issues, ethics committees and data protection commissioners should be more actively involved to establish concrete operational rules that, once followed, guarantee compliance with data protection requirements. This would also allow to keep up with the new ethical and legal questions generated by the increasing availability of novel forms of health data (e.g., those generate by fitness devices or mobile applications). Although ethics committees can have difficulties in approaching innovative projects involving processing of health-related data (67) and cantonal data protection commissioners struggle with underfunding (68), it is necessary for data processors (e.g., researchers) to coordinate with these actors, who are *de facto* in charge of applying the law on health data processing.

Compliance with consent norms and data protection rules when re-using data would also be facilitated by reducing the fragmentation of procedures to access the different sources of health information (69). This can be achieved in several ways: for example, by securing that every institution storing health data possesses a clear access-point that external stakeholders can easily find and contact, if they wish to collect data from that source. Else, common and shared requirements could be established, indicating what the necessary procedures (e.g., security requirements, permission necessary by cantonal data protection officers, etc.) are for accessing data in a legally and ethically compliant fashion. Efforts in this sense have already started, with the drafting of legal agreement templates by the SPHN,^13^ aimed at standardising the documentation necessary for data-exchange between institutions. However, the use of such documentation was primarily designed for the exchange of data between *academic* institutions, thus excluding important sources of health data in Switzerland, such as health insurances. Such documents could thus be further developed as to become easily usable also in other contexts. The creation of a comprehensive national data center could also contribute to this aim, as it could offer a unique transit-station, where all bureaucratic steps necessary to access data from different institutions could be channelled and more efficiently solved. Such center should also be adequately structured and financed, so that its functioning is expedite and efficient.

### Next Steps and Knowledge Transfer

The findings of this study add to the evidence produced recently in Switzerland to suggest the way forward for the health data landscape [see e.g. (45)], which is one of the main priorities in the vision for the Swiss healthcare in the next decade (18). The further step for our research team is to present the findings to a group of stakeholders during a workshop, in order for them to reflect on the recommendations, refine them and – if possible – find a consensus on the main priorities for the future of the Swiss context. The insights presented in this study and in the project where it is embedded (29) are also going to inform the knowledge transfer activities of the National Research Program (NRP) 74 (70). The NRP 74 was launched in 2015 on initiative of the Swiss Government and the Swiss National Science Foundation to fund projects aiming at “making healthcare smarter” and then selected 34 projects - including the one where this study belongs - to help reach this aim. To ensure that the research by NRP 74 funded projects reaches policymakers and other relevant stakeholders, the NRP 74 created a synthesis process aimed at summarising and condensing the evidence produced by all 34 selected projects (71, 72). Such synthesis process includes a specific section on the topic of health data, into which our team is feeding the insights produced by our research. This will lead to the creation of policy briefs that will be delivered to relevant stakeholders and discussed at a final symposium planned for mid-2022 (71).

### Limitations

This study has some limitations. Firstly and more importantly, it includes the view of a plurality of stakeholders, but it does not consider other important actors which are involved in the governance of health data in Switzerland – such as data protection commissioners, health insurance companies and the public. This limitation was due to choices made for the design of the project where this study is nested and it thus requires to integrate its findings with those of other studies considering the perspectives of different stakeholders [for citizens' perspectives in the use of data, see e.g., (73, 74)]. Also, participants were selected non-randomly, which underlines the non-generalizability of our findings. Moreover, we cannot exclude that some of the responses in our interviews were influenced by social desirability, especially considering that participants were aware that this study was part of a project by an institute specialised in biomedical ethics.

## Conclusion

Improving the health data framework of a country is a lengthy and long-term endeavour with no silver-bullet solutions. It is however a worthy endeavour, since a solid and well-functioning health data infrastructure is an important element for evidence-based policymaking and for appropriate public health interventions. With this study, we presented and analysed the inputs from stakeholders who have an interest in improving the situation in Switzerland and who are thus motivated to find solutions that are both effective, but also practical. We have explored the proposed recommendations and have discussed their feasibility, showing that progress cannot be revolutionary, but rather evolutionary, in that new proposals have to be reconciled with the pre-existing infrastructural, legal and ethical backgrounds. Rather than a swift change, a gradual development of the health data framework appears preferable. Our study is thus particularly useful as a reference to steer policymaking at a national level. However, it is also an important source of information for other countries that are transitioning toward a more digitalised healthcare and that might profit from the experience of Switzerland and from the recommendations expressed by our stakeholders. Countries which are further ahead in the development of an effective system of health data exchange also obtain a competitive advantage for their health system and their researchers, as the case of Denmark shows (75). Learning from the experiences of nations that are successful in improving health data usage as well as nations which still face challenges is equally important. Indeed, for achieving progress in each single country, it is necessary to find the appropriate compromise between the system of health data exchange that researchers and public health practitioners ideally desire, the preferences of society at large and the pre-existing data infrastructure and organisation of healthcare services.

## Data Availability Statement

The raw data supporting the conclusions of this article will be made available by the authors, upon reasonable request to the corresponding author. Full transcripts cannot be shared to keep the risk of re-identification of participants low, as it was guaranteed to the interviewees upon participation.

## Ethics Statement

Ethical review and approval was not required for the study on human participants in accordance with the local legislation and institutional requirements. Written informed consent for participation was not required for this study in accordance with the national legislation and the institutional requirements.

## Author Contributions

BSE and TW conceived the study and prepared the interview guide with LDG and AM. Data were collected by AM and LDG. AM, TW, and SME prepared the first draft of the manuscript. BSE, LDG, and FE integrated the initial draft with comments and additions to the manuscript. FE provided additional review concerning the legal and policy aspects. AM finalised the last version of the manuscript, which was then further corrected, and approved by all authors. All authors contributed to the article and approved the submitted version.

## Conflict of Interest

FE is affiliated with the Swiss Institute of Bioinformatics (SIB), a non-profit organization predominantly publicly funded that is dedicated to biological and biomedical data science and that collaborates with the SPHN. The remaining authors declare that the research was conducted in the absence of any commercial or financial relationships that could be construed as a potential conflict of interest.

## Footnotes

^1^See the Human Research Act. Available online at: https://www.fedlex.admin.ch/eli/cc/2013/617/en (accessed January 29, 2021).

^2^Segments were translated to English internally by our research team, which features proficient users (C1 and C2 proficiency according to the Common European Framework of Reference for Languages - CEFR) of all languages used (English, French, German, and Italian).

^3^The abbreviation gives details about the interviewee. The letters indicate from which group of participants the interview of the coded segment belongs. “Res” refers to researchers, “Pol” to policymakers and “Stak” to directors or administrators of an institution managing health data. The number refers to the order of the interview within that group of stakeholders. So, for example “Res20” means: 20th Interview conducted with a Researcher as interviewee.

^4^Other care providers (such as private medical practices) have the option of offering the EPD, but no legal obligation to do so.

^5^See https://santeperso.ch/A-propos (accessed April 15, 2021).

^6^See https://datascience.ch/who-we-are/ (accessed April 15, 2021).

^7^See https://www.midata.coop/en/cooperative/ (accessed April 15, 2021).

^8^For more information see the websites of these two institutions: https://www.nacr.ch/ or https://www.nicer.org/ (accessed January 29, 2021).

^9^For more details on this service offered by the FSO, see the dedicated page at: https://www.bfs.admin.ch/bfs/de/home/dienstleistungen/datenverknuepfungen/fuer-dritte.html (accessed January 29, 2021).

^10^For more details, see the dedicated page at: https://sphn.ch/network/projects/data-coordination-center/the-sphn-semantic-interoperability-framework/ (accessed February 10, 2021).

^11^For more details see the dedicated page on the: https://www.zas.admin.ch/zas/it/home/partenaires-et-institutions-/unique-person-identification–upi-/identifiant-du-dossier-electronique-du-patient.html (accessed January 29, 2021).

^12^The Swiss federal council recently suggested that research using EPD data could be performed according to Swiss law on human research [https://www.parlament.ch/de/ratsbetrieb/suche-curia-vista/geschaeft?AffairId=20194136 (accessed January 29, 2021)], but this possibility remains controversial.

^13^These are available at: https://sphn.ch/services/dtua/ (accessed January 29, 2021).

## Abbreviations

EPD: Electronic Patient Dossier
UPI: Unique Patient Identifier
FOPH: Federal Office of Public Health
SPHN: Swiss Personalized Health Network
FSO: Federal Statistical Office.
