# Supplementary material for: Evolution or Revolution? Recommendations to Improve the Swiss Health Data Framework
Source: Front Public Health. 2021 May 31;9:668386. doi: 10.3389/fpubh.2021.668386 (PMC8200489; doi:10.3389/fpubh.2021.668386)
Supplement: Supplementary file 1 [file Table_1.DOCX]

# **Interview Guide – SMAASH NRP-74 project**

*Let me begin by asking you some questions on your professional/research domain.*

Q1. Could you please walk me through your professional/research activity in relation to health data?

Q2. Could you tell me more on your most recent project which currently involves the collection and sharing of health data?

Q3. Could you tell me more about these data collections you are using? Which institution provided

them?

What additional types of data are being used?

Can you please describe how you acquired such data?

Q4. What is your opinion on data sharing for your project?

Q5. Have you experienced barriers towards the acquisition and/or sharing of such data?

What were these barriers and how were they addressed?

Q6. If using multiple data collections (referring to Q3):

Concerning the databases/registries you are currently working with, how did you manage to link these data sources to your data?

Q7. Have you experienced OR are you anticipating barriers towards the analysis of those data for your

project? Could you elaborate more on these challenges? How were these addressed?

*I would like now to switch gears and move towards legal and ethical considerations concerning data collection and data sharing and would love to learn your perspectives on those:*

Q8. Do you consider informed consent for data collection/sharing? What is your strategy for obtaining

it or justification for not obtaining it?

Q9. For your project, did you ever experience any legal/regulatory challenges? What were these

challenges?

Did you abide to any existing national/international regulatory and ethical guidelines pertaining to your professional/research activities? If yes, which ones? How did they influence your project?

Do you see any room for improvement and if yes, what exactly?

Q10. We usually hear that institutions as well as individual researchers are not keen in sharing health

data. What is your opinion on this?

Q11. In the context of your project, do you feel comfortable sharing the data you collected directly or after the first analyses, and can you explain why?

In your opinion, under what conditions would third parties be allowed to use your data?

If you agree that there are conditions under which third parties can gain access, who should these third parties be?

Q12. In light of our interesting discussion, do you have any specific recommendations you would like

to make which will help to improve the health data situation in Switzerland?

Q13. Do you have any question or comment that you would like to add before we end our discussion?

*It has been a pleasure knowing more about you and your research/professional activities in regard to health data. We thank you for your participation and time.*
